# Stress and Withdrawal from Chronic Ethanol Induce Selective Changes in Neuroimmune mRNAs in Differing Brain Sites

**DOI:** 10.3390/brainsci6030025

**Published:** 2016-07-27

**Authors:** Darin J. Knapp, Kathryn M. Harper, Buddy A. Whitman, Zachary Zimomra, George R. Breese

**Affiliations:** 1 Bowles Center for Alcohol Studies, The University of North Carolina at Chapel Hill, CB7178, Chapel Hill, NC 27599-7178, USA; Kathryn_harper@med.unc.edu (K.M.H.); bawhitman@gmail.com (B.A.W.); zzimomra@kent.edu (Z.Z.); george_breese@med.unc.edu (G.R.B.); 2 Department of Psychiatry, The University of North Carolina at Chapel Hill, CB7178, Chapel Hill, NC 27599-7178, USA; 3 Curriculum in Neurobiology, The University of North Carolina at Chapel Hill, CB7178, Chapel Hill, NC 27599-7178, USA; 4 Department of Pharmacology, The University of North Carolina at Chapel Hill, CB7178, Chapel Hill, NC 27599-7178, USA; 5 UNC Neuroscience Center, The University of North Carolina at Chapel Hill, CB7178, Chapel Hill, NC 27599-7178, USA

**Keywords:** restraint stress, chronic ethanol withdrawal, cytokine mRNAs, CRF, alcohol, CP154,526

## Abstract

Stress is a strong risk factor in alcoholic relapse and may exert effects that mimic aspects of chronic alcohol exposure on neurobiological systems. With the neuroimmune system becoming a prominent focus in the study of the neurobiological consequences of stress, as well as chronic alcohol exposure proving to be a valuable focus in this regard, the present study sought to compare the effects of stress and chronic ethanol exposure on induction of components of the neuroimmune system. Rats were exposed to either 1 h exposure to a mild stressor (restraint) or exposure to withdrawal from 15 days of chronic alcohol exposure (i.e., withdrawal from chronic ethanol, WCE) and assessed for neuroimmune mRNAs in brain. Restraint stress alone elevated chemokine (C–C motif) ligand 2 (CCL2), interleukin-1-beta (IL-1β), tumor necrosis factor alpha (TNFα) and toll-like receptor 4 (TLR4) mRNAs in the cerebral cortex within 4 h with a return to a control level by 24 h. These increases were not accompanied by an increase in corresponding proteins. Withdrawal from WCE also elevated cytokines, but did so to varying degrees across different cytokines and brain regions. In the cortex, stress and WCE induced CCL2, TNFα, IL-1β, and TLR4 mRNAs. In the hypothalamus, only WCE induced cytokines (CCL2 and IL-1β) while in the hippocampus, WCE strongly induced CCL2 while stress and WCE induced IL-1β. In the amygdala, only WCE induced CCL2. Finally—based on the previously demonstrated role of corticotropin-releasing factor 1 (CRF1) receptor inhibition in blocking WCE-induced cytokine mRNAs—the CRF1 receptor antagonist CP154,526 was administered to a subgroup of stressed rats and found to be inactive against induction of CCL2, TNFα, or IL-1β mRNAs. These differential results suggest that stress and WCE manifest broad neuroimmune effects in brain depending on the cytokine and brain region, and that CRF inhibition may not be a relevant mechanism in non-alcohol exposed animals. Overall, these effects are complex in terms of their neuroimmune targets and neuroanatomical specificity. Further investigation of the differential distribution of cytokine induction across neuroanatomical regions, individual cell types (e.g., neuronal phenotypes and glia), severity of chronic alcohol exposure, as well as across differing stress types may prove useful in understanding differential mechanisms of induction and for targeting select systems for pharmacotherapeutic intervention in alcoholism.

## 1. Introduction

Acute withdrawal from chronic ethanol (WCE) exposure is associated with increased anxiety-like behavior [1,2,3]. Breese et al. [4] also found in a three-withdrawal protocol that stress substituted for the initial two withdrawals such that withdrawal from a single five-day cycle of chronic ethanol induced anxiety. Subsequently, Breese et al. [5] found that the anxiety-like response to restraint stress was facilitated when the stress was applied after WCE—a finding in agreement with other reports that stress after WCE can enhance negative effects [6,7].

Breese et al. [8] and Knapp et al. [9] also reported that administration of lipopolysaccharide (LPS) or a cytokine into the brain substituted for the initial intermittent ethanol exposures applied prior to a single CE exposure to induce negative effects. This latter outcome was comparable to the change observed with prior exposure to stress [4,5] or after WCE [2,3,10]. More recently, Whitman et al. [9] reported that WCE increased cytokine mRNAs in the cortex—a cytokine immune response that was not related to infection [11,12,13]. Whitman et al. [9] also observed increases in mRNAs for toll-like receptor 4 (TLR4) and High Mobility Group Box 1 Protein (HMGB1) which serve as an endogenous system that activates neuroimmune function [11,12,14,15,16]. The induction of cytokine mRNAs increases after WCE was blocked by a CRF1 receptor (CRF1R) antagonist [9]—a finding possibly linking the cytokine mRNA changes to CRF involvement in the anxiety-like behavior that accompanies WCE and stress [3,4,5]. 

Various studies have linked stress to increases in cytokine mRNAs in various brain sites [17,18,19,20,21,22,23,24,25]. Collectively, these reports provided new information about the effects of various stressors (social stress/defeat, footshock or tailshock, restraint, forced swim, glucose/insulin challenge, or cold stress) on neuroimmune mRNA responses of the hypothalamus, hippocampus, cerebellum, posterior cortex, and nucleus of the solitary tract. Relatedly, work from our group had shown that restraint stress could substitute for the initial repeated exposures to chronic alcohol to induce a negative emotional state following a future withdrawal as inferred from anxiety-like behavior, (e.g., [4]). To explore the potential relevance of stress effects on neuroimmune responses in a chronic ethanol and withdrawal model, Breese et al. [8] administered LPS or a pro-inflammatory cytokine into brain to substitute for the initial intermittent ethanol withdrawals or mild stress to induce anxiety-like behavior following a single ethanol withdrawal that otherwise would be incapable of eliciting anxiety [4,5]. Missing from this strategy was an assessment of whether the restraint stress itself induced neuroimmune changes consistent with functional effects on behavior. Thus, a key new component of the current studies was to assess whether stress, which by itself has been shown in some studies to increase brain cytokines (e.g., [18,20,21,26]), produced changes comparable to those triggered by WCE. Additionally, comparisons were made of cytokine mRNA in different brain regions after stress or WCE. Finally, to complement earlier studies with WCE and cortical cytokines, the present study explored whether a corticotrophin-releasing factor receptor antagonist would attenuate stress-induced cytokines in the cortex. This line of inquiry is pertinent to understanding the relative overlap of neuroimmune effects of stress and the WCE model in relation to alcohol abuse, drug addiction, and several psychiatric disorders (see [6,7,8,27,28,29,30,31,32,33,34,35]).

## 2. Materials and Methods

### 2.1. Animals 

Adult male Sprague–Dawley (S–D) rats or Wistar Rats (Charles-River, Raleigh, NC, USA) weighing 180–200 g upon arrival were group housed and fed RMH3000 rat chow (Test Diets, Richmond, IN, USA) for 2–3 days prior to study to acclimate them to the new environment (temperatures 70–72 °F; humidity 40%–60%; and light/dark cycle 12 h:12 h with lights from 7:00 a.m. to 7:00 p.m.). Subsequently, all rats were singly housed for the duration of experimental procedures. Methods used in this study were approved by Institutional Animal Care and Use Committee (IACUC, protocol number: 14-125) at the University of North Carolina (Chapel Hill, NC, USA). 

### 2.2. Liquid Diet for Controls and for Chronic Ethanol Exposure

**Figure 1:**
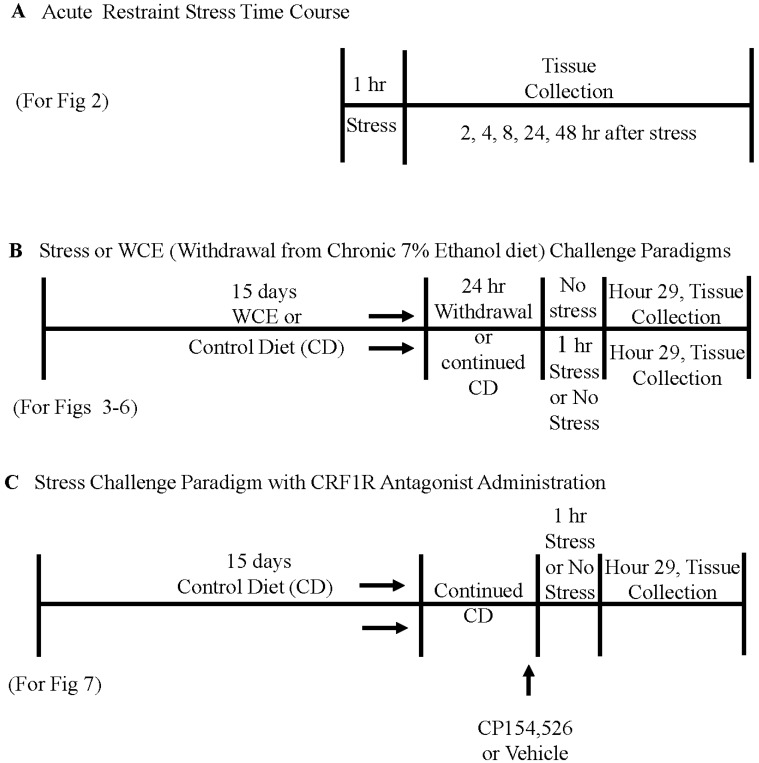
Schematics depicting experimental protocols. **A**: represents the acute restraint stress time course, while **B**: represents the stress and withdrawal from chronic alcohol and **C**: represents the CRF1R antagonist study.

In the initial experiment, rats that received an acute restraint stress were on chow diet before the stress challenge. For other experiments, a nutritionally-complete and calorically-balanced liquid diet was used for the rats that received a continuous 7% (*w*/*v*) ethanol diet followed by 24 h of withdrawal (WCE)—an approach previously utilized by our laboratory (e.g., [1,36,37]; see Figure 1) to administer daily ethanol doses of 9–13 g/kg. The liquid control diet (CD) was calorically balanced to the WCE diet by adjustments of the amount of dextrose. Rats were fed either control diet or the ethanol diet with a modified pair-feeding strategy [1]. 

### 2.3. Restraint Stress in Controls and after Withdrawal from Chronic Ethanol Exposure

Initial efforts determined the time course of stress effects on brain cytokine mRNAs. Stress consisted of 60 min of restraint in plastic decapicones. These rats were sacrificed 2, 4, 8, 24, or 48 h following the stress (see schematic in Figure 1A). 

### 2.4. Brain Tissue Collection and Real-Time PCR Analysis for Tissue mRNA

Following experimental procedures, rats were rapidly decapitated and brain tissue stored at −80 °C for subsequent extractions for PCR. To initiate PCR procedures, total RNA was extracted with Trizol (Invitrogen, Carlsbad, CA, USA) from homogenized dissected brain regions from control and ethanol-treated experimental brain sections followed by use of the SV total RNA isolation system (Promega, Madison, WI, USA). This tissue was then used for reverse transcription PCR using the Superscript First Strand or Superscript III First Strand Synthesis Super mix (Life Technologies, Grand Island, NY, USA) [38]. The primer sequences used for the cortex were chemokine (C–C motif) ligand 2 (CCL2) = 5′-TCACGCTTCTGGGCCTGTTG-3′ (forward) and 5′-CAGCCGACTCATTGGGATCATC-3′ (reverse); Interleukin-1β (IL-1β) = 5′-GAAACAGCAATGGTCGGGAC-3′ (forward) and 5′-AAGACACGGGTTCCATGGTG-3′ (reverse); tumor necrosis factor-α (TNFα) = 5′-ATGTGGAACTGGCAGAGGAG-3′ (forward) and 5′-ACGAGCAGGAATGAGAAGAGG-3′(reverse); Toll-like-receptor-4 (TLR4) = 5′-GCCGGAAAGTTATTGTGGTGGT-3′ (forward) and 5′-ATGGGTTTTAGGCGCAGAGTTT-3′ (reverse); β-actin, 5′-ATGGTGGGTATGGGTCAGAAGG-3′ (forward) and 5′-GCTCATTGTAGAAAGTGTGGTGCC-3′ (reverse). SYBR green PCR master mix (Applied Biosystems, Foster City, CA, USA) was used for real-time PCR analysis of the cortex on the Bio-Rad MyiQ (Bio-Rad, Hercules, CA, USA). For other brain regions (and the CP154,526 study), mRNA analyses were optimized with TaqMan^®^ (Thermo Fisher Scientific, Waltham, MA, USA) expression assays—CCL2 (Rn00580555_m1), TNFα (Rn01525859_g1), IL-1β (Rn00580432_m1), TLR4 (Rn00569848_m1), and β-actin (Rn00667869_m1)—and samples were run on a StepOnePlus real time PCR machine (Life Technologies, Grand Island, NY, USA). For all data, the cycle time (Ct) values were normalized with β-actin to assess the relative differences in expression between groups. Ct values of β-actin never differed across groups therefore β-actin was an appropriate choice as a housekeeping gene. Calculated values were expressed as relative change to a designated control set as 100%.

### 2.5. Enzyme-linked Immunosorbent Assay (ELISA) for Cytokines

**Figure 2:**
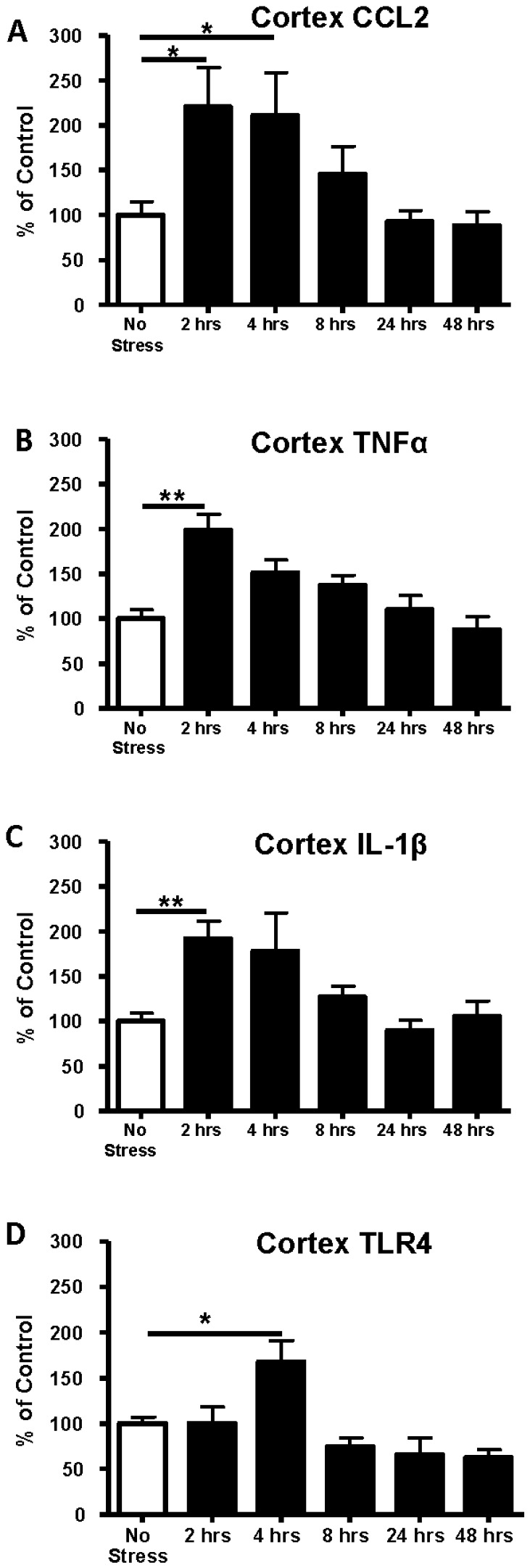
Time course of changes in cerebro-cortical mRNAs for CCL2, IL-1β, TNFα, and TLR4 following 1 h of restraint stress. Stress elevated CCL2 mRNA in cortex (*F*(5,32) = 3.82, *p* = 0.008), an effect that peaked at 2 and 4 h. A similar effect was found for IL-1β mRNA (*F*(5,48) = 5.13, *p* < 0.01) and TNFα (*F*(5,53) = 8.51, *p* < 0.0001). TLR4 mRNA was elevated at 4 h (*F*(5,38) = 8.5, *p* < 0.0001). In all cases, the cytokine mRNA levels gradually returned to control levels by 24 h. ** *p* < 0.01 * *p* < 0.05 compared with controls that received no stress (open bars). **A**, **B**, **C**, and **D** delineate data for CCL2, TNFα, IL-1β and TLR4 mRNAs, respectively, (*n* = 5–8 per time point)

Because changes in levels of cytokine proteins in the S–D rats have been found to correlate poorly with mRNA changes (see [38,39]) initially only expression of mRNAs for cytokines was assessed. Nonetheless, because of interest in the relationship between mRNA and proteins induced by stress, ELISA assays for cytokine proteins in cortex were first performed 4 h after stress in the time course determination in the S–D rats (Figure 2). Subsequently, assays of cytokine proteins were performed 4 h after an acute restraint stress to Wistar rats (Charles River, Raleigh, NC, USA). Each cortical sample was homogenized in Iscove’s Modified Dulbecco Medium (Invitrogen, #12440046, Carlsbad, CA, USA) containing 1 tablet per 50 mL of the complete protease inhibitor cocktail (Roche Diagnostics #11697498001, Indianapolis, IN, USA). Homogenized specimens were then centrifuged at 12,000× *g* for 10 min at 4 °C and the supernatants collected and stored at −80 °C until the ELISA determination was made. ELISA kits were purchased for IL-1β and TNFα from R & D Systems, (Minneapolis, MN, USA), and for the CCL2 from BD Bio-Sciences (San Jose, CA, USA). ELISA procedures were performed according to the manufacturer’s instructions. Standards for IL-1β and TNFα were serially diluted 4 times to concentrations of 1.95 pg/mL and 0.78 pg/mL, respectively, and the standard for CCL2 was used as supplied. All tissue cytokine levels were corrected for protein using Pierce^®^ BCA Protein assay (Thermo Scientific, Rockford, IL, USA). 

### 2.6. CRF Receptor Antagonist Administration

Subgroups of CD rats were injected once with the CRF1R antagonist CP154,526 [38,40] or vehicle 15 min prior to the start of stress (Figure 1C). The drug was prepared as a microfine suspension in 0.5% carboxymethylcelluose and administered intraperitoneally at a dose of 15 mg/kg in a volume of 2 mL/kg.

### 2.7. Statistical Analysis

Data (expressed as mean ± standard error of mean (SEM)) were evaluated for statistical significance with ANOVA with Fisher’s least significant difference (LSD) tests for individual comparisons of group pairs as appropriate. Individual data points that were three standard deviations from their respective group means were removed from the group prior to analysis. *p*-values < 0.05 were considered statistically significant. 

## 3. Results

### 3.1. Time Course of Expression of Cytokine and TLR4 mRNAs in Cortex Following 1-Hour of Restraint Stress in Sprague–Dawley (S–D) Rats

Previous studies have shown that acute stress can affect neuroimmune function in brain [13,14,18,19,20,21,22,24,25,26,41]. Therefore, our initial investigation was to determine if the restraint stress utilized in previous behavioral studies [5] would induce a neuroimmune response. Figure 1A shows the experimental protocol for determining cytokine mRNA changes after 60 min of restraint stress in the absence of WCE. As shown in Figure 2, each mRNA assayed was increased 2–4 h after the restraint stress. The expression of CCL2 mRNA after stress was significantly increased above control by 121% at 2 h and by 111% at 4 h (*p* < 0.05). The expression of TNFα mRNA after stress was increased above control by 2 h (99%) (*p* < 0.01). Likewise, IL-1β mRNA was elevated by 92% above control by 2 h (*p* < 0.01). Cytokine mRNAs gradually returned to control levels by 8 h and remained there for up to 48 h after the acute-stress exposure (Figure 2). Because TLR4 has been implicated in induction of cytokines [11,15,41,42,43,44,45], we also examined whether mRNA for TLR4 would be altered by the acute restraint-stress. Figure 2D shows that TLR4 mRNA expression was significantly elevated by 68% above control 4 h following the stress challenge (*p* < 0.05) with return to control levels by 8 h.

### 3.2. Determination of Cytokine Protein Levels in Cortex after Restraint Stress

To determine whether increases in cytokine proteins accompanied the increases in CCL2, IL-1β, and TNFα mRNAs induced by restraint stress, proteins were measured in cortex 4 h after the restraint stress challenge to the S–D rats (Table 1). Cytokine protein levels, unlike cytokine mRNAs, in the controls were not statistically altered in the S–D rats (Table 1). Subsequently, this same assessment was performed for Wistar rats to determine if this rat strain might express a change in cytokine proteins following the 60 min of restraint stress. In the Wistar rats, as in the S–D rats, cytokine proteins were not increased by stress (*p* > 0.05). Because increases in cytokine protein levels were not observed in either rat strain [38], only S–D rats were used in the experiments assessing expression of cytokine mRNAs induced by stress or WCE.

### 3.3. Effect of Stress or WCE on Selected Cytokine and TLR4 mRNAs in Cortex

**Figure 3:**
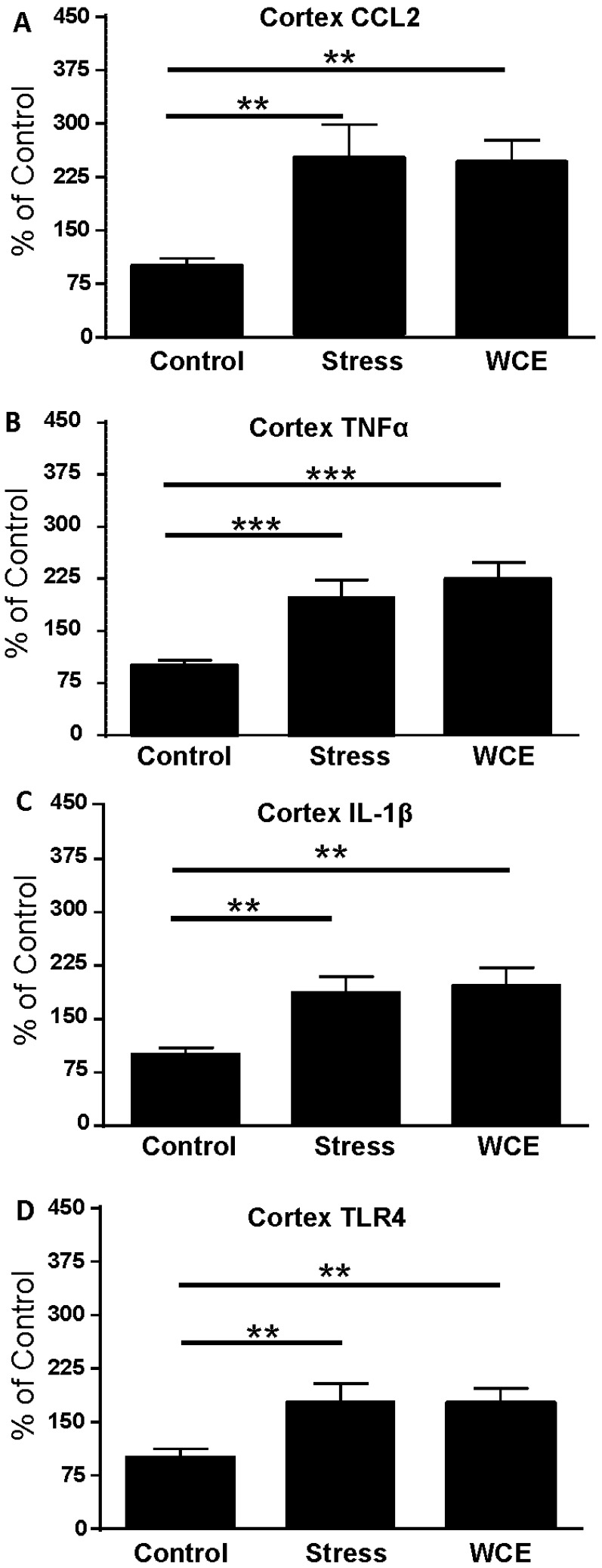
Effects of restraint stress or withdrawal from chronic ethanol (WCE) on cerebro-cortical neuroimmune mRNAs. Control and stress rats received non-ethanol containing liquid diets and 1 h restraint stress or no stress, respectively. WCE rats received chronic ethanol liquid diet for 15 days followed by 29 h of withdrawal. Overall, a significant effect was also noted across the groups for CCL2 (*F*(2,23) = 6.47, *p* < 0.01) with individual comparison of groups revealing significant effects of stress or WCE relative to controls. A similar profile was noted for TNFα (*F*(2,32) = 10.2, *p* < 0.001), IL-1β (*F*(2,27) = 7.00, *p* < 0.01), and TLR4 (*F*(2,31) = 4.97, *p* < 0.05) and individual group comparisons to the respective controls. * *p* < 0.05, ** *p* < 0.01, ** *p* < 0.001 versus Controls. **A**, **B**, **C**, and **D** delineate data for CCL2, TNFα, IL-1β and TLR4 mRNAs, respectively, (*n* = 8–12 per group).

In prior work, WCE increased anxiety-like behavior [46] and elevated cortical cytokines [38]. This study directly compared the magnitude of the WCE effect on cytokine mRNA with that produced by stress. Figure 3 shows that an acute 60 min restraint stress increased cortical cytokine mRNAs 4 h later to a degree comparable to that observed in Figure 2. Likewise, in accord with previous work [38], Figure 3 confirms that CCL2, IL-1β, TNFα, and TLR4 mRNAs were significantly increased over controls in cortex 29 h after WCE (*p* < 0.05). These cytokine mRNA increases following WCE were comparable in magnitude (80%–150% over control) to the increases induced by stress (Figure 2).

### 3.4. Effect of Stress or WCE on Selected Cytokine mRNAs in Hypothalamus, Hippocampus, and Amygdala after WCE

**Figure 4:**
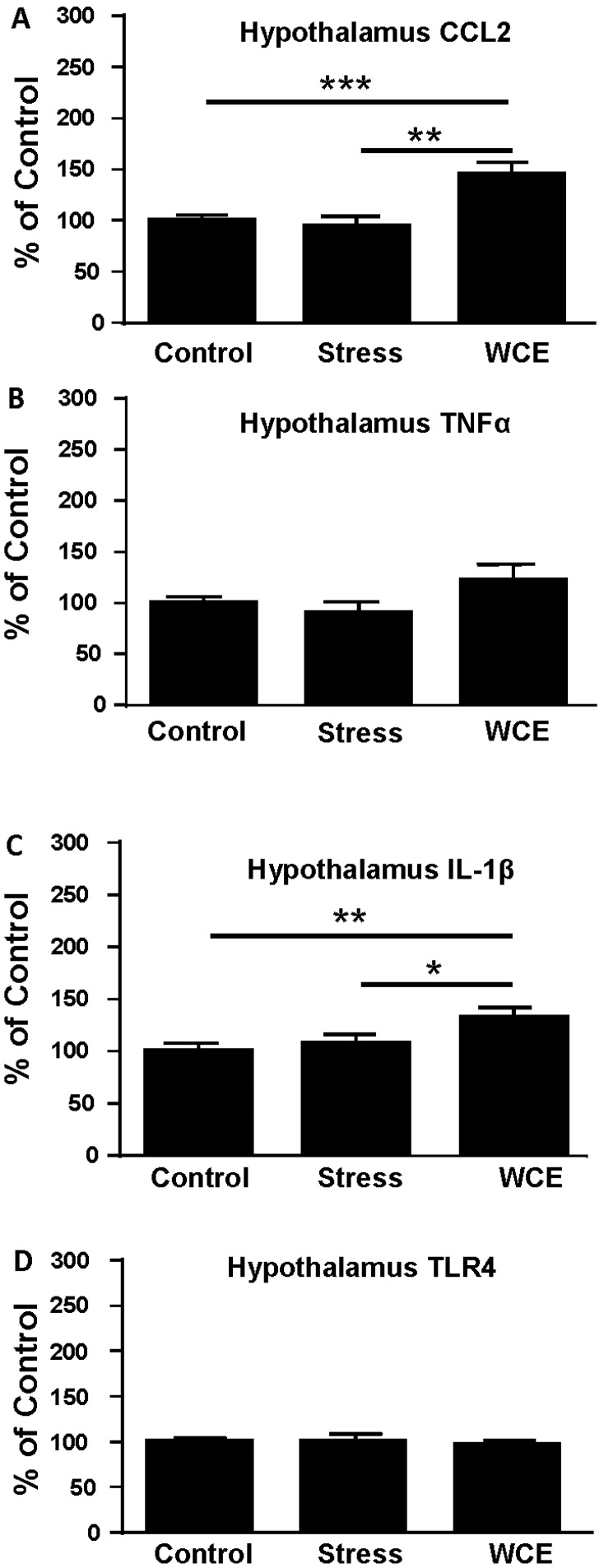
Effects of restraint stress or WCE on hypothalamic neuroimmune mRNAs. For CCL2, there was a significant overall effect of treatments (*F*(2,35) = 9.13, *p* < 0.001) with a significant group comparison relative to controls revealed only with the WCE treatment. For TNFα, there was no overall effect of treatment (*p* > 0.05) despite a trend toward a WCE effect. There was an overall effect of treatment on IL-1β (*F*(2,38) = 4.59, *p* < 0.05) with the WCE group being higher than controls or stressed rats. Finally, there were no effects on TLR4. Group designations are the same as those in Figure 3. * *p* < 0.05, ** *p* < 0.01, ** *p* < 0.001 versus controls. **A**, **B**, **C**, and **D** delineate data for CCL2, TNFα, IL-1β and TLR4 mRNAs, respectively, (*n* = 8–13 per group).

To examine the generalizability of the cortical neuroimmune mRNA response, the effects of stress or WCE were studied in additional brain regions of known importance in chronic ethanol effects. Figure 4 illustrates that, whereas stress did not increase CCL2 or IL-1β mRNA in the hypothalamus (*p* > 0.05, Figure 4A,C), WCE increased both of these cytokine mRNAs significantly (by about 30%–45%, *p* < 0.01). Further, hypothalamic TLR4 and TNFα mRNAs were not significantly altered by either challenge (*p* < 0.05 vs. control).

**Figure 5:**
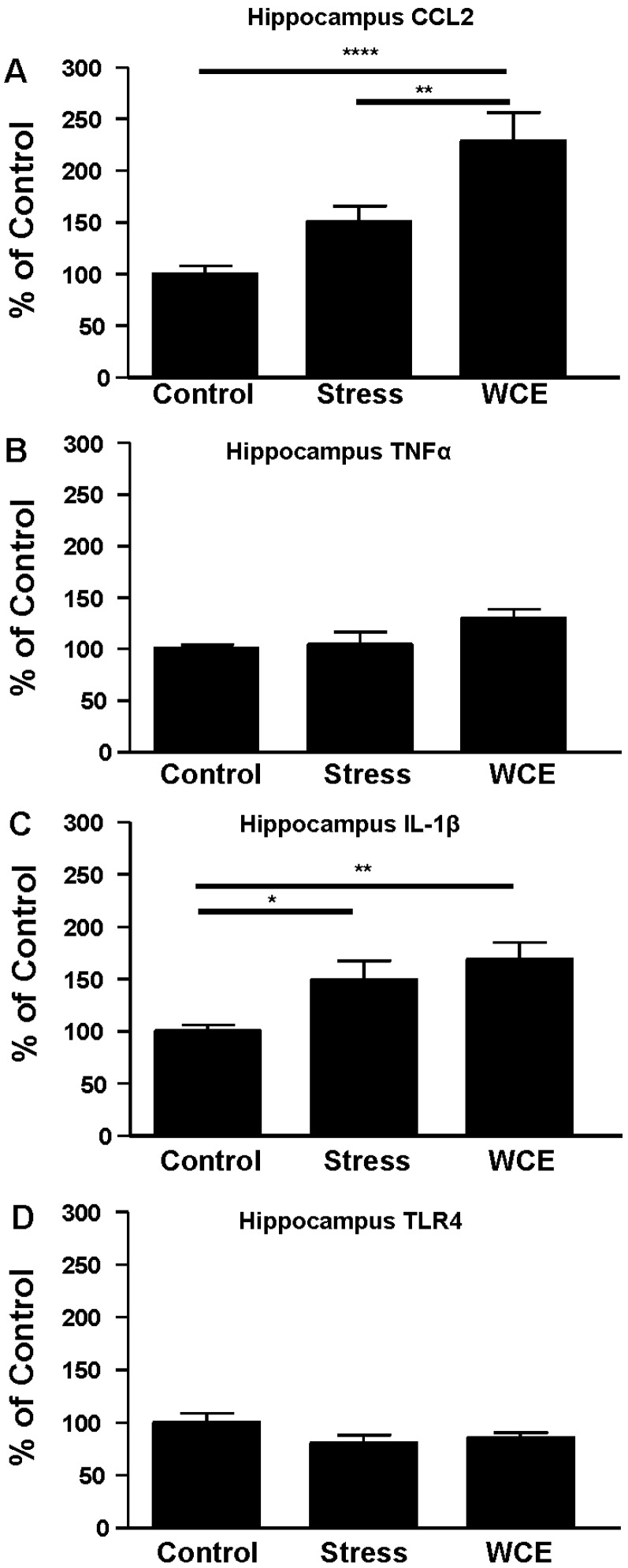
Effects of restraint stress or WCE for hippocampal neuroimmune mRNAs. For CCL2, there was an overall effect of the treatments (*F*(2,34) = 10.54, *p* < 0.001) and a significant effect of WCE relative to stress or control. The trend toward a stress effect was not significant. Similarly, an overall trend toward a significant effect of treatments for TNFα was not significant (*p* = 0.07). For IL-1β, there was an overall significant effect of treatments (*F*(2,34) = 4.98, *p* < 0.05) with significant effects of stress and WCE relative to controls. Finally, there were no significant effects found with TLR4 mRNA. * *p* < 0.05, ** *p* < 0.01, **** *p* < 0.0001 versus Controls. **A**, **B**, **C**, and **D** delineate data for CCL2, TNFα, IL-1β and TLR4 mRNAs, respectively, (*n* = 9–13 per group).

**Figure 6:**
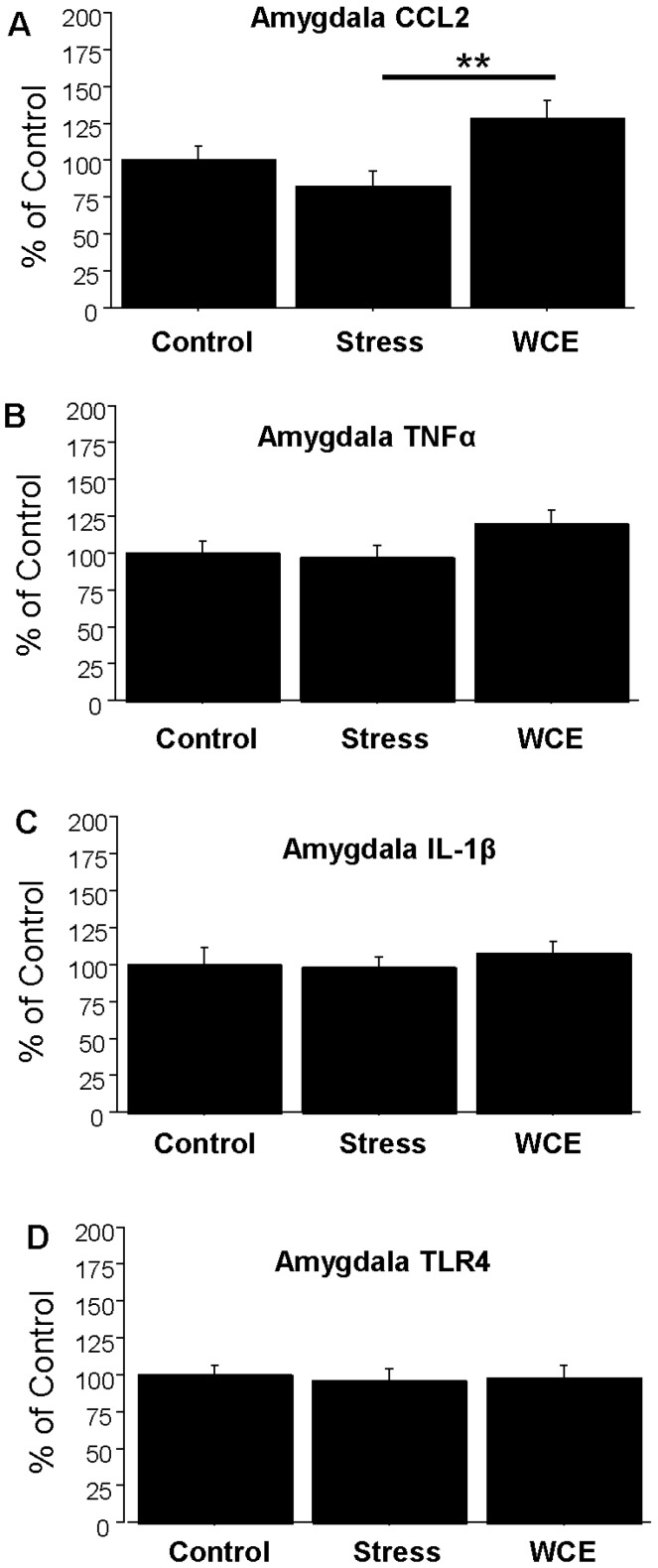
Effects of restraint stress or WCE on amygdala neuroimmune mRNAs. For CCL2, there was a modest, but significant, overall effect of treatments (*F*(2,27) = 4.450, *p* < 0.05) with a significant group difference between WCE and stress. There were no other effects of treatments for TNFα, IL-1β, or TLR4 mRNAs. ** *p* < 0.01 compared with the stressed group. **A**, **B**, **C**, and **D** delineate data for CCL2, TNFα, IL-1β and TLR4 mRNAs, respectively, (*n* = 7–11 per group).

In the hippocampus, CCL2 (Figure 5A) increased with stress (by approximately 50%), but the effect of WCE was more dramatic (129% of control) and significantly higher than that for stress. In contrast, while WCE tended to increase TNFα in this region, no significant effects were found in response to stress (Figure 5B), with a similar result for TLR4 (Figure 5D). With regard to IL-1β (Figure 5C), both stress and WCE comparably induced this cytokine. Finally, in the amygdala (Figure 6), there was no effect of stress alone on any measure (*p* > 0.05), although WCE significantly increased CCL2 relative to stress levels. 

### 3.5. Effect of the CRF1R Antagonist CP154,526 on Cortical Cytokine mRNAs Following Stress

**Figure 7:**
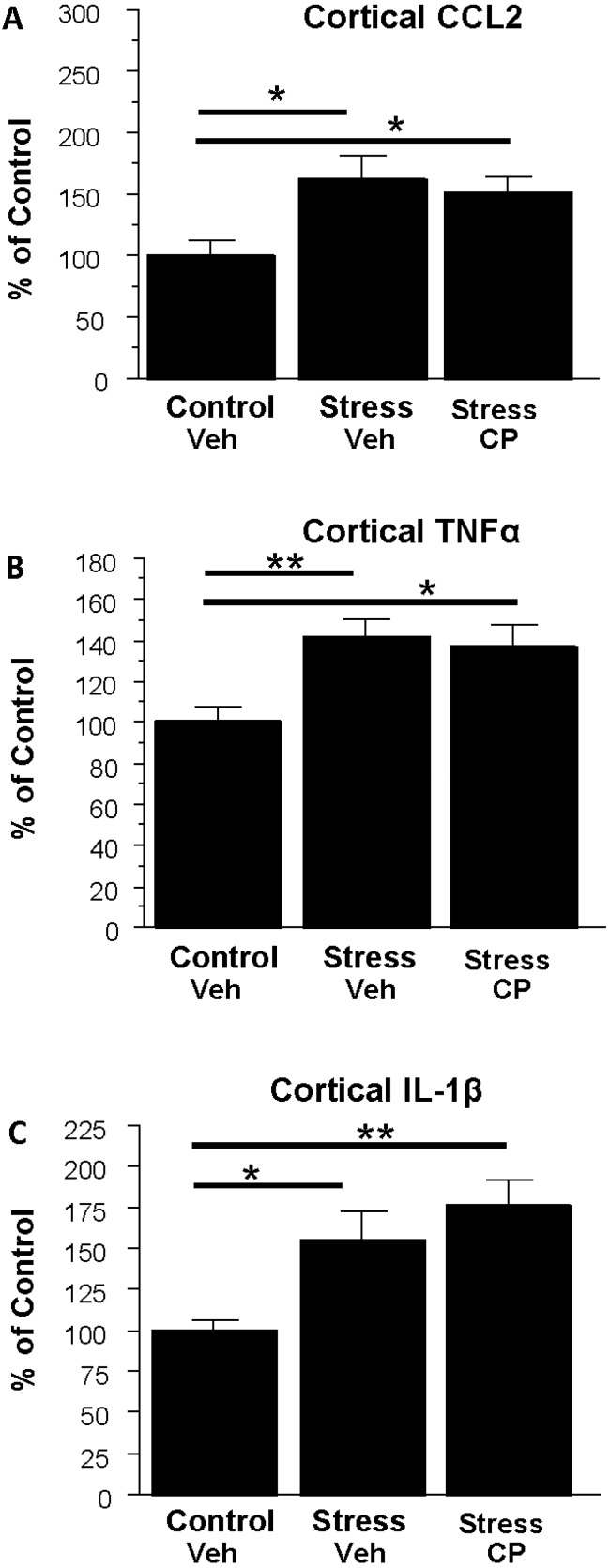
Effects of the corticotropin-releasing factor 1 (CRF1R) antagonist CP154,526 (CP) on cerebro-cortical neuroimmune mRNAs following restraint stress in rats. Overall there was a significant effect among the groups for CCL2 (*F*(2,16) = 4.00, *p* < 0.05) with CP failing to block stressed-induction. There also was an effect of treatments for TNFα (*F*(2,16) = 4.87, *p* < 0.05) with stress inducing and CP failing to block induction. Finally, as with CCL2 and TNFα, cortical IL-1β effects were significant overall (*F*(2,16) = 6.34, *p* < 0.01), but there was no blockade of the stress effect by the drug. * *p* < 0.05, ** *p* < 0.01 versus control-vehicle treated rats. Veh = 0.5% carboxymethylcellulose. **A**, **B**, and **C** delineate data for CCL2, TNFα, IL-1β and TLR4 mRNAs, respectively, (*n* = 5–7 per group).

Having twice shown previously that at CRF1R antagonist blocks cytokine induction arising during ethanol withdrawal in the cortex [38], the final experiment focused on determining whether the drug would also block the induction due to stress. Figure 7 illustrates the effect of CP154,526 on cytokine mRNAs in the cortex. Figure 7A shows that stress increased CCL2 (63%, *p* < 0.05) and this effect was not blocked by CP154,526. Similarly, Figure 7B shows that TNFα was increased by stress (41%, *p* < 0.01) and again the stress effect was unaltered by CP. Finally, the profile of action on IL-1β was similar as shown in Figure 7C which shows that IL-1β was significantly increased by stress (55%, *p* < 0.05) and CP154,526 failed to block this induction.

## 4. Discussion

In the present investigation, restraint stress increased the mRNAs for CCL2, IL-1β, and TNFα and the receptor TLR4 in cortex of S–D rats (Figure 2 and Figure 3)—a result supporting a growing body of reports of acute stressor-induced increases in neuroimmune mRNAs in various brain sites [19,20,23,24,26,47]. The restraint stress-associated increase in cytokine and TLR4 mRNAs in cortex peaked by around 4 h after exposure and then returned to control levels within 1 day. However, stress did not always affect these mRNAs in other brain sites. Even though mRNAs were increased 4 h after stress exposure in controls, corresponding cytokine proteins were not altered at this time point in either S–D or Wistar Rats (Table 1). Neuroimmune responses to WCE sometimes followed the response to stress and sometimes did not. While CCL2, TNFα, IL-1β, and TLR4 responses in the cortex were generally comparably high with either challenge, responses in the amygdala were comparably minimal. However, in the hippocampus, responses varied by type of challenge and across neuroimmune markers with a robust CCL2 response to withdrawal and a comparable IL-1β response to either challenge. In the hypothalamus, stress was inactive while withdrawal elevated CCL2 and IL-1β. Finally, the results showed that CRF1R inhibition did not alter stress-induced neuroimmune responses in the cortex, a result inconsistent with the earlier findings that CRF1R inhibition blocked cytokine responses to chronic withdrawal [38].

The reason that stress increased select cytokine mRNAs but not corresponding cytokine proteins is unknown. This pattern of mRNA versus protein results is similar to that observed in studies in other areas of rat brain. For example, Hueston et al. [21] demonstrated an increase in IL-1β mRNA without an accompanying increase in IL-1β protein in the paraventricular nucleus (PVN) to restraint stress. Deak et al. [19] found that IL-1β protein did not increase in the hypothalamus of S–D rats after restraint stress alone but did observe an increase in IL-1β to stress in the hypothalamus after applying a combination of restraint and shaking (i.e., a more severe stress). Additionally, Porterfield et al. [39] found that 2 h of restraint increased expression of IL-1β mRNA in the hypothalamus and caused a corresponding increase in this cytokine protein in the more stress-responsive Fischer 344 rats, but not in S–D rats [39]. However, Whitman et al. [38] found that an acute LPS challenge increased both selected cytokine proteins and corresponding cytokine mRNAs in the cortex of S–D rats [38]. While a focus on the hypothalamus and IL-1 (e.g., [19,23]) has been a fruitful focus of prior research so far as consistent effects of stress are concerned, reports of combinations of stressors may be particularly worthy of follow up. In addition to the work of Deak et al. [19] and Porterfield et al. [39] noted above, prior cold stress rendered animals’ neuroimmune systems responsive to future LPS treatment [20], as shown by elevated hypothalamic and prefrontal cortical neuroimmune markers). Considered in aggregate, such studies suggest that genetic background and/or the degree and combinations of stress or challenge to the neuroimmune system may be at least in part responsible for the presence of a neuroimmune response and possibly for differences in protein versus mRNA responses as well. It may be particularly interesting in this context to examine the possibility that differentially engaged molecular mechanisms of mRNA versus protein processing across time may account for asynchrony of these constructs. That is, the observation of a stress-associated increase in cytokine mRNAs without corresponding changes in cytokine proteins could also be explained by release, utilization, and degradation of the protein during the stress challenge. Relatedly, habituation or exhaustion of mRNA generation could also be a factor in some cases. In this context, Minami et al. [23] showed that the IL-1β mRNA response in the hypothalamus declined over four hours despite continued immobilization stress. Such an effect could conceivably have influenced our amygdala and hypothalamic findings, but would be harder to extend to our cortical and hippocampal results. Also relevant is the more recent report of Vecchiarelli et al. [26] who found that increasing the length of restraint stress in Sprague–Dawley rats to two hours elevated protein levels of amygdala TNFα, decreased IL-6, while monocyte chemoattractant protein-1 (MCP-1/CCL2) remained unchanged. Based on our data, it is unlikely that any such diminished cortical response would apply to the cortex globally. Differential responses across subregions of the cortex might be critical with the net effect of global cortical responses being an increased response that must be dependent on some region(s) being particularly sensitive. Extending this logic to the hippocampus or hypothalamus may be premature, as Vecchiarelli did not see changes in these regions. The single hour of restraint stress in the present work did not alter amygdala or hypothalamic TNFα mRNA but did increase cortical IL-1β, TNFα and CCL2, and hippocampal IL-1β. Perhaps the length or severity of stress, along with a focus on cortical subregions represent key prerequisites to examinations of either individual or combinatorial challenges. Further research to explore these various possibilities is warranted, including combinations of different intensities, times, and types of stress with chronic alcohol challenges, circadian rhythms, and genetic background. 

To assess the possible contribution of other components to neuroimmune activation pathways that result in the effects observed here, TLR4 mRNA was also assessed after restraint stress. A significant increase in TLR4 mRNA, was observed only at 4 h in cortical tissue following stress, returned to control level by 8 h, and remained at this level through 48 h after the stress (Figure 2D). This fairly tight time-response relative to the other mRNAs was one of the reasons for focusing on 29 h of withdrawal (4 h post stress relative to non-stressed alcohol withdrawn rats) as the target for comparison. A consideration here is that prior assessments [38] focused mostly on alcohol withdrawal-derived mRNA data from the 24th hour of withdrawal although they showed that mRNAs can remain elevated for longer periods, thus it seems unlikely that mRNA levels would be meaningfully different across these two time points within the present study. Regardless, these results agree with Blandino et al. [18] who found no change in cortical TLR4 mRNA at their 2 h time point post footshock or LPS treatment. It is notable that stress or WCE elevated TLR4 mRNA only in the cortex. The reasons for this specific effect on TLR4 are unknown, but suggests differential neuroimmune regulation and possibly lower thresholds for activation across brain regions. The TLR4 receptor complex is a prominent driver in neuroimmune processes in general and in alcoholism (e.g., see [48]) and animal models in particular (e.g., [38,49,50]). In fact, the TLR4 receptor may play a role in a positive feedback loop that amplifies the intensity of overall neuroimmune activation in alcoholism [51]. Such reduced thresholds for neuroimmune activation may be represented most profoundly following endotoxin where activation progresses to neurodegeneration in the substantia nigra with corresponding behavioral deficits [52].

While WCE alone generally elevated cortical cytokines (and see [38]) and hippocampal CCL2 and IL-1β, WCE or stress alone did not consistently do so for some mRNAs in some regions (e.g., the hippocampus and hypothalamus, Figure 4 and Figure 5). The limited TNFα response in these regions contrasts with the effects of pain-associated stress such as footshock reported by Blandino et al. [18] and thus supports the idea that the type of stress may be important in cytokine induction. Again, the pattern of results suggests that unique mechanisms are operating across brain regions and it would likely be productive to further examine neuroimmune mRNAs more generally on a region by region basis and thus speak to the relatively understudied yet critical issue of how neuroimmune changes across regions or networks could produce neuropathology. The potential differential induction of anti-inflammatory cytokines such as IL-10 could also be informative. These future studies should elucidate how stress induction of some cytokine mRNAs, but not others, contributes to the profile of neuroimmune activation in models of alcoholism [3,4,5,9].

One potentially important focus in identifying mechanisms of cytokine regulation in this experimental context relates to the corticotropin-releasing factor (CRF) system. The data herein show that the CRF1R antagonist CP154,526 was inactive against stress induction of neuroimmune mRNAs. This effect provides an interesting comparison with previous work [38] that focused on the effect of this drug on cytokine mRNAs elevated by WCE. The mechanisms that explain how the drug comes to exert an effect on one challenge and not the other are unknown, but differential engagement of adaptive mechanisms might be one possibility. That is, the drug may affect a recruited process unique to rats experiencing WCE. This interesting possibility is reminiscent of the work of Koob and colleagues who noted that CRF receptor antagonist effects were generally not manifest unless rats were dependent on alcohol (e.g., [53]). It is important to note that the CP154,526 study herein was limited in its scope and could be expanded in future studies to examine related questions. For example, the drug could be employed in examining the effect on cytokine mRNAs in the context of combined stress and WCE which is arguably a very relevant experience in some alcohol abusers. 

While the current research corroborates the demonstration by Whitman et al. [38] that cytokine mRNAs are increased in the cortex 24 h after WCE and extends our inquiry into other brain sites and to the effects of stress, the studies do not address this potentially interesting combinatorial effects of the two challenges. What our results do show is that effects of these challenges are each themselves complex and perhaps more nuanced in their consequence on the neuroimmune system than may have previously been appreciated. Such findings prompt considerations of the work of others where it was shown that the neuroimmune system may respond differently to stress depending on the degree and nature of prior challenges [17,20]. In related research, Buck et al. [54] reported that a single dose of ethanol before foot-shock stress had no effect on immune function and did not enhance the stress-induced increase in IL-1β mRNA in the PVN. In general, a second challenge before or after the WCE would seem appropriate strategy to gain new evidence for the possibility that an initial challenge to the neuroimmune system may permit or alter induction of select neuroimmune mediators by a second challenge. Thus, sufficient previous activation of immune function by chronic ethanol exposure might render stress capable of further increasing cytokine mRNAs, as previously noted behaviorally [2,4,10,55]. Thus, identification of the conditions under which prior stress or chronic alcohol exposure alters future responses to either challenge would seem to be a productive avenue for research.

Whatever future combinatorial stress studies might reveal, the present results nonetheless do provide an interesting contrast with Whitman et al. [38], who demonstrated that a CRF1R antagonist prevented the cytokine mRNA increases induced by the WCE alone. It would also be of interest to identify differential physiological effects of the drug in context of the two challenges. For example, in considering the idea that cytokines may have specific neurophysiological and behavioral actions manifest in select brain regions (e.g., [9,56]), it would seem likely that broadening the neuroanatomical focus of these CRF/cytokine interactions would very likely be a productive endeavor (see also [57]). Collectively, these studies implicate CRF involvement in the increased expression of cytokine mRNAs during the 24 h withdrawal from the WCE and suggest that there may be functional consequences. In this regard, amygdala CRF-amplified CCL2 regulation of alcohol self-administration [58] and elevated CRF-dependent amygdala CCL2 in human alcoholics [48] are consistent with a role of cytokines and CRF interacting to regulate alcohol consumption. These findings considered in the context of chronic alcohol dependent CCL2 induction within the central amygdala and robust elevations of the TNF receptor (Tnfrsf1a) in rats, support the idea that neuroimmune mechanisms in the amygdala are potentially critical in the behavioral pathology in alcoholism [59]. Thus, a future experiment should be undertaken to further examine the interactions of stress and WCE across additional relevant brain regions and to further isolate relevant mechanisms. Likewise, based upon the report by Johnson et al. [22] that norepinephrine-receptor antagonists blocked the stress increase in hypothalamic IL-1β protein induced by inescapable tail shock (i.e., a severe stress), and the finding that the beta-adrenergic agonist isoproterenol can enhance IL-1 production in the amygdala following chronic stress (Porterfield et al., [39]), the effects of this drug class on the cytokine mRNA changes across different brain regions after restraint stress in the presence and absence of WCE should also be explored.

## 5. Conclusions

The present findings provide additional evidence for neuroimmune involvement in brain function associated with stress or WCE and the differential induction of neuroimmune mRNAs and adds the novel observation that a CRF1R antagonist is inactive against a mild stress. The results herein show that some neuroimmune components are readily inducible in a brain-region-dependent manner while others are not. Such evidence adds to a growing literature that implicates neuroimmune dysfunction in alcoholism, other substance abuse disorders, and other neurobehavioral disorders associated with stress [7,9,27,28,32,60,61]. Our findings and others prompt questions about how some challenges exert specific neuroimmune effects within neuroanatomically limited areas and suggest further studies should be done to examine combinations of challenges/conditions thought to impact on alcoholism and associated neuropsychiatric conditions. Moreover, our findings, considered in the context of the documented roles of the neuroimmune system and stress in alcohol consumption and negative emotional symptoms due to chronic ethanol consumption [9,58,62,63,64,65], support the idea that specific neuroimmune processes are engaged in neurobehavioral processes fundamental to alcohol abuse. It may be productive to ask whether risk of relapse in alcoholics relates to differential neuroimmune responses to stress as a consequence of prior chronic ethanol exposure history and to further develop animal models around this concept. These findings also have potentially broader implications in that neuroimmune system dysfunction has been implicated in other neurobehavioral disorders as well including insomnia [33], depression [31,34,66,67,68], and anxiety [6,35,60,69]. In particular, neuroimmune system regulation of anxiety associated with chronic alcohol and withdrawal are notable [8,9,49]. Identifying overlapping and independent neuroimmune processes across these pathologies would be worthy of further study. 

While the current studies document that neural mechanisms associated with stress may at least partially overlap with the mechanisms that drive cytokine mRNA expression after WCE, the profile of effects shown herein for the two challenges do not completely overlap across neuroimmune marker or brain region. Further, the responses reported herein were elicited with relatively limited challenges (i.e., just 1 h of stress or 15 days of exposure to ethanol). Thus, it may be valuable to examine similar endpoints following exposure to the more chronic and/or severe challenges/stressors that define many neurobehavioral disorders. Of these effects, one could ask which are transient (yet perhaps behaviorally relevant), and which induce long term maladaptations that influence behavioral pathology. By understanding how stress and WCE engage the neuroimmune system, and worsens symptoms, therapeutic options by which to mitigate stress-associated neuroimmune dysfunction in drug addiction and other central nervous system disorders could emerge [29,32].

## Figures and Tables

**Table 1:** Effect of acute stress on cytokine proteins in brain.

Group	CCL2	IL-1β	TNFα
S-D Non-Stressed	12.9 (0.3)	0.37 (0.05)	ND
S-D Stressed	13.7 (0.6)	0.28 (0.02)	ND
Wistar Non-Stressed	42.2 (2.3)	1.48 (0.22)	0.11 (0.03)
Wistar Stressed	58.1 (15.4)	1.05 (0.84)	0.12 (0.03)

Data are mean +/− standard error of mean (SEM) protein/mg total protein from cerebral cortex of rats that were restrained for 1 h and sacrificed 4 h later. CCL2: Chemokine (C–C motif) ligand 2; IL-1β: Interleukin-1-beta; TNFα: Tumor Necrosis Factor alpha; ND: not detectable; S–D: Sprague–Dawley.

## Abbreviations

ANOVA	analysis of variance
CCL2	chemokine (C-C motif) ligand 2
CRF	corticotropin releasing hormone
IL-1β	interleukin-1 beta
mRNA	messenger ribonucleic acid
S-D	Sprague-Dawley
TLR4	toll-like receptor 4
TNFα	tumor necrosis factor-alpha
WCE	withdrawal from chronic ethanol
qPCR	quantitative polymerase chain reaction
